# Multidisciplinary management of antiepileptic drug-induced toxic epidermal necrolysis in a young woman

**DOI:** 10.7705/biomedica.7581

**Published:** 2025-09-22

**Authors:** Salman Khan, Priti Singh, Rana Salieva

**Affiliations:** 1 Department of Pathological Processes and Therapeutics, American University School of Medicine Aruba, Oranjestad, Aruba American University School of Medicine Aruba Oranjestad Aruba; 2 Department of Premedical Sciences, and Nutritional, Biochemical and Molecular Sciences, American University School of Medicine Aruba, Oranjestad, Aruba American University School of Medicine Aruba Oranjestad Aruba; 3 Pulmonology, Osh Interregional Joint Clinical Hospital, Osh, Kyrgyzstan Osh Interregional Joint Clinical Hospital Osh Kyrgyzstan

**Keywords:** Stevens-Johnson syndrome, hypersensitivity, Síndrome de Stevens-Johnson, hipersensibilidad

## Abstract

Toxic epidermal necrolysis is a rare, life-threatening dermatological emergency characterized by extensive epidermal detachment and mucosal involvement, associated with high morbidity and mortality. Early diagnosis and prompt treatment are imperative to improving patient outcomes.

This case report describes the clinical course, management, and outcomes of a 29-year-old female diagnosed with toxic epidermal necrolysis. She had a history of polypharmacy and medication allergies and presented with a sudden-onset fever, malaise, and a diffuse rash. Clinical examination revealed extensive epidermal detachment involving more than 80% of the body surface area, including mucous membranes. Carbamazepine, administered recently, was identified as the suspected causative agent.

The patient was promptly admitted to the intensive care unit for specialized care, including supportive measures, wound care, and close monitoring of fluid and electrolyte balance. Intravenous immunoglobulin therapy was initiated, together with a multidisciplinary approach involving dermatology, ophthalmology, and nutritional support. The patient's condition gradually improved over the following weeks, with re-epithelialization of the affected areas and resolution of systemic symptoms.

This case highlighted the importance of early diagnosis, prompt management, and a multidisciplinary approach in optimizing patient outcomes. As toxic epidermal necrolysis is a rare disease, prospective studies, immunogenetic biomarkers, and randomized controlled trials remain limited. Further research in these areas is needed to provide valuable insights into the management of this disease.

## Resumen

La necrólisis epidérmica tóxica es una emergencia dermatológica poco común y potencialmente mortal, caracterizada por desprendimiento epidérmico extenso y afectación de las mucosas, asociada con una elevada morbilidad y mortalidad. El diagnóstico temprano y el tratamiento oportuno son fundamentales para mejorar los resultados clínicos. Este reporte de caso presenta el curso clínico, el manejo y el resultado de una paciente diagnosticada con necrólisis epidérmica tóxica. Se trata de una mujer de 29 años con antecedentes de polifarmacia y alergias a medicamentos, que presentó fiebre de inicio súbito, malestar general y un exantema difuso. En el examen clínico se observó desprendimiento epidérmico extenso que afectaba más del 80 % de la superficie corporal, incluyendo las mucosas. La carbamazepina, administrada recientemente, fue identificada como el agente causal sospechoso.

La paciente fue ingresada de inmediato en la unidad de cuidados intensivos para recibir atención especializada, que incluyeron medidas de soporte, cuidado de heridas y un estricto control del equilibrio de líquidos y electrolitos. Se inició terapia con inmunoglobulina intravenosa y el caso fue abordado con un enfoque multidisciplinario que involucró dermatología, oftalmología y soporte nutricional. La condición clínica de la paciente mejoró gradualmente durante varias semanas, con reepitelización de las áreas afectadas y resolución de los síntomas sistémicos.

Este caso resalta la importancia de un diagnóstico temprano, manejo oportuno y enfoque multidisciplinario para optimizar los desenlaces de esta enfermedad. Dado que la necrólisis epidérmica tóxica es una afección poco frecuente, no existen estudios prospectivos, biomarcadores inmunogenéticos ni suficientes ensayos clínicos aleatorizados de referencia. Se requiere más investigación en estas áreas para generar conocimientos que aporten al manejo de esta enfermedad.

Toxic epidermal necrolysis constitutes a severe and potentially life-threatening dermatological condition characterized by extensive epidermal detachment, mucous membrane involvement, and a spectrum of systemic manifestations. In 1956, Alan Lyell described this phenomenon as "a skin eruption resembling scalding" ^(^^1^. This condition is a rare and significant adverse drug reaction, frequently triggered by pharmacological agents. Toxic epidermal necrolysis has an estimatedannual incidence ranging from 0.4 to 1.2 cases per million individuals ^2^^-^^4^. Its precise pathogenesis remains unknown, but it appears to represent an immune-mediated hypersensitivity reaction induced by pharmacological agents ^3^^,^^5^. The drugs frequently implicated in the onset of this disorder include antibiotics, particularly sulfonamides and cephalosporins; antiepileptic medications, such as carbamazepine and phenytoin; and nonsteroidal anti-inflammatory drugs (NSAID), like ibuprofen and allopurinol ^4^^-^^6^.

Toxic epidermal necrolysis typically begins with flu-like symptoms, followed by a prodromal phase characterized by fever, malaise, and skin tenderness. This phase progresses to the emergence of extensive erythematous macules that swiftly evolve into bullae and subsequent epidermal detachment ^4^^,^^7^^,^^8^. Involvement of the mucous membranes is common, mainly affecting the oral, ocular, and genital areas ^5^^,^^7^^,^^9^.

Management of toxic epidermal necrolysis requires prompt cessation of the causative pharmacological agent and provision of supportive care within an intensive care unit setting. Supportive interventions encompass fluids and electrolytes management, wound care, pain alleviation, nutritional support, and infection prevention. The administration of systemic corticosteroids may be employed, although their efficacy remains controversial ^7^^,^^9^^-^^11^. Other therapeutic approaches, including intravenous immunoglobulin and cyclosporine, have shown encouraging results in select cases ^8^^,^^12^.

This case report describes the clinical manifestation, management, and outcome of a patient diagnosed with toxic epidermal necrolysis.

## Case presentation

The patient, a 29-year-old female, arrived at the emergency department in an exceedingly critical state characterized by widespread cutaneous eruptions, heightened anxiety, cephalalgia, hyperthermia reaching 40 °C, and conjunctivitis. Her medical history included outpatient treatment for high fever and migraines at a private healthcare center, where she was prescribed multiple drugs, including carbamazepine, NSAID, and vitamin B. After undergoing infusion therapy, her condition deteriorated, requiring admission to the intensive care unit.

### Status at admission

Upon admission, the patient's overall health status was critical, primarily due to the toxic repercussions of pharmacotherapy. She remained conscious, exhibiting a normosthenic body composition, and assumed a passive posture.

**Figure 1:**
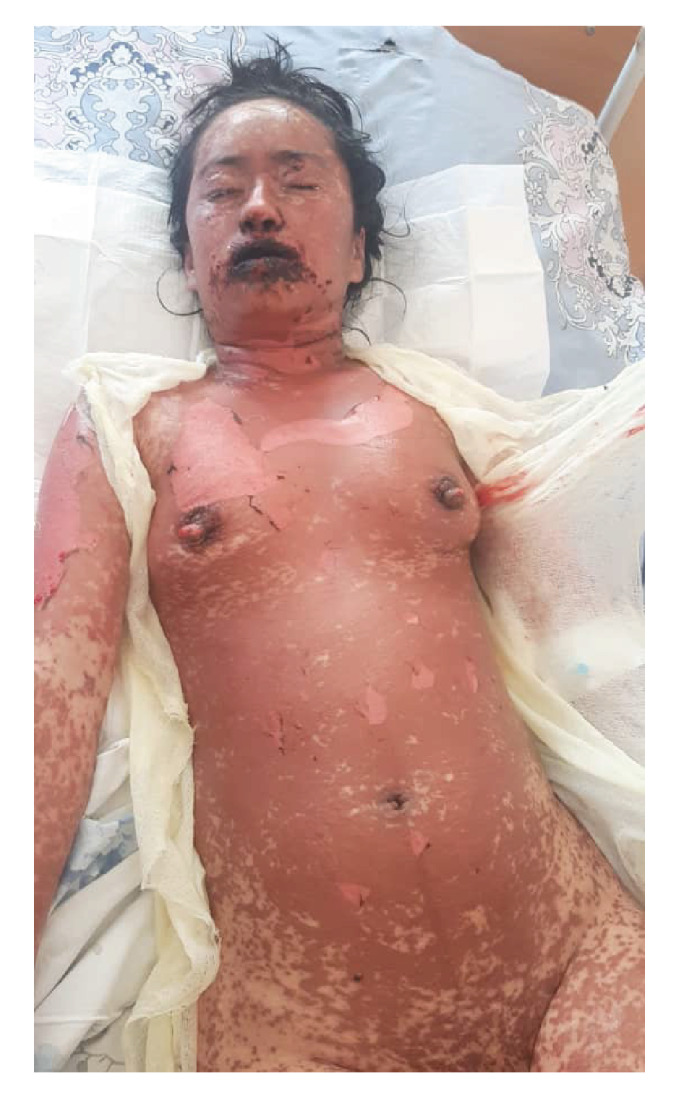
Confluent dusky macules and epitelial detachment involving the face, lips, chest, and abdomen. Blistered lips with erythematous mucous membranes. Eyes with epitelial erosions and ocular erythema.

**Figure 2:**
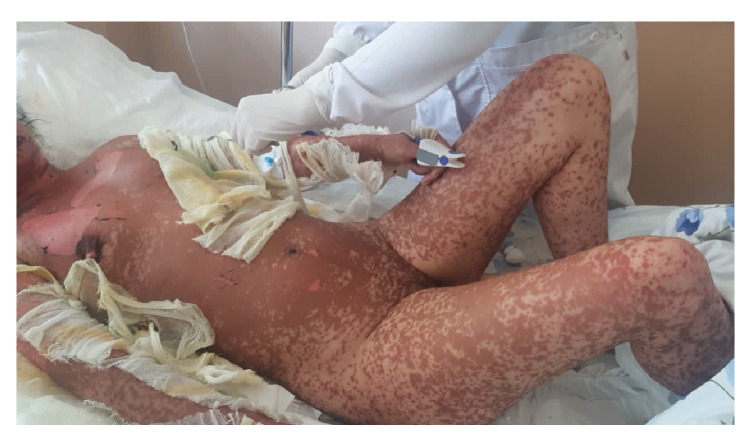
Maculopapular erythematous eruptions with scattered bullae involving approximately 36% of the body surface area.

**Figure 3:**
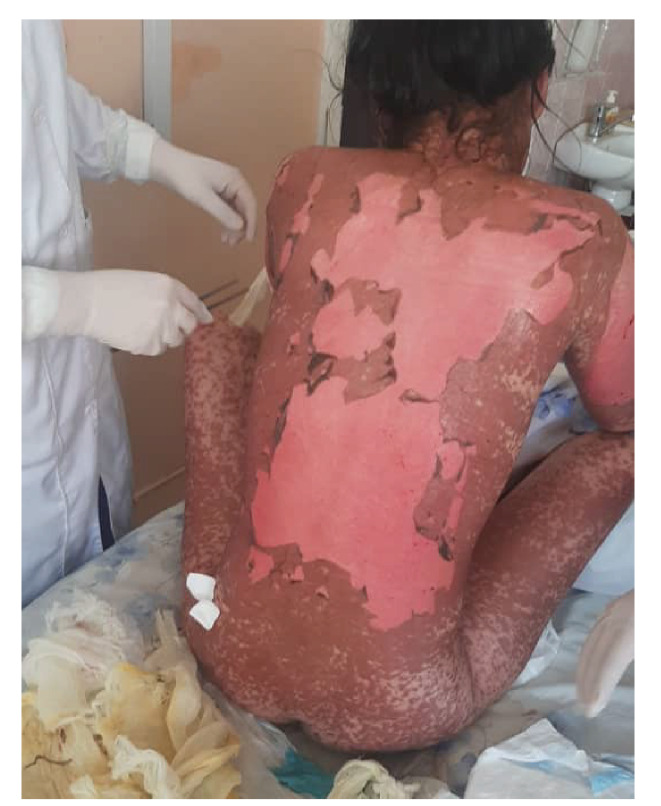
Posterior body surface showing maculopapular erythematous eruptions with epithelial detachment and bullae.

**Figure 4:**
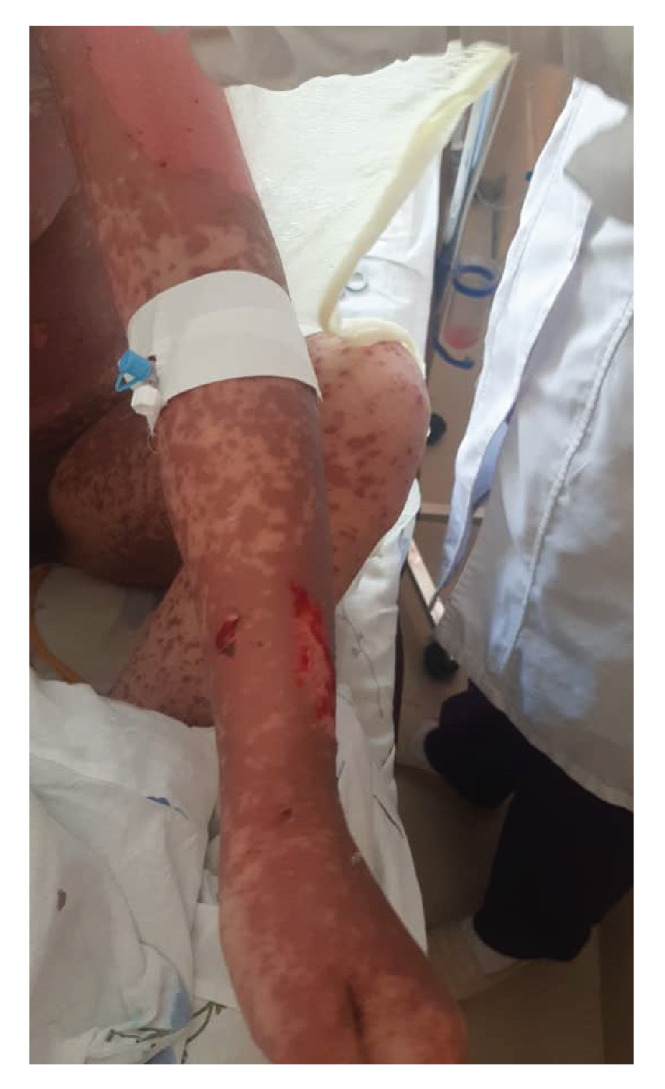
Maculopapular erythematous eruptions with epithelial detachment and bullae on the upper extremity.

Her integumentary system appeared pallid, with edematous, painful, erythematous lesions of different dimensions localized on the torso, extremities, face, oral mucosa, and genitalia. Notably, she had dusky coalesced macules and epithelial detachment on the face, lips, thorax, abdomen, back, and upper and lower extremities; also, she presented with vesicular formations within the nasal and oral cavities (figures 1-4).

The patient exhibited vesicular breath sounds without wheezing. Her heart rate was 112 beats per minute, and her blood pressure was 120/80 mm Hg; her lingual surface was dry and coated. The abdominal examination revealed a soft non-tender abdomen. Stool output and diuresis were within normal ranges.

### Laboratory findings

The complete blood count (CBC) revealed hemoglobin at 104 g/L, 3.61 * 10^12^ erythrocytes/L, 100 * 10^9^/L platelets, 3.00 * 10^9^ leukocytes/L, 78.0 * 10^9^ neutrophils/L, - 18.2 * 10^9^ lymphocytes/L, and 3.8 * 10^9^ monocytes/L. The erythrocyte sedimentation rate (ESR) was at 50 mm/h, and hematocrit levels were at 30.0%. Hemostatic evaluation showed_a prolonged partial thromboplastin time (PTT) of 40.3 s, a markedly prolonged activated partial thromboplastin time (APTT) higher than 2 minutes, and a fibrinogen level of 8,888 mg/L. The ethanol precipitation test was negative.

Liver function tests showed mild hyperbilirubinemia (total = 10.3 μmol/L, direct = 2.3 μmol/L, and indirect = 8.0 μmol/L), elevated transaminases (alanine aminotransferase, ALT = 427 IU/L; aspartate aminotransferase, AST = 555 IU/L) 13,14, Thymol turbidity test of 0.93, and reduced serum protein (55.4 g/L).

Biochemical analysis revealed elevated nitrogen (22.80 mmol/L), urea at 7.52 mmol/L, creatinine of 66.7 jimol/L, and glucose at 4.19 mmol/L. Protein fractions showed albumin at 33.4 g/L, gamma-globulins at 29.6 g/L, total protein of 63.0 g/L, and an albumin/globulin ratio of 1.1.

Rheumatologic tests indicated elevated antistreptolysin (ASLO = 1:400 IU/ml), C-reactive protein at 1:54 IU/ml, rheumatoid factor at 1:64 IU/ml, and Waaler-Rose test at 1:24 IU/ml. Immunologic evaluation showed high levels of immunoglobulin E (234.9 IU/ml). The patient's blood type was B (III), Rh-positive. Urinalysis revealed the presence of a high number of erythrocytes and leukocytes.

### Treatment

The patient was immediately admitted to the intensive care unit for management. Supportive measures included fluid resuscitation, pain control, wound care, and prevention of secondary infections. An ophthalmological consultation was required to manage conjunctivitis.

The patient started on systemic corticosteroids with 1 mg/kg/day of methylprednisolone. Additional supportive care included intravenous fluids (300 ml of physiological solution, 200 ml of Ringer's solution), a proton pump inhibitor (40 mg of omeprazole), antihistamines (2 ml of suprastin administered intramuscularly), adsorbent therapy (enteros gel), topical treatment (advant ointment), and antimicrobial agents (200 ml of Ciprox and 150 mg of fluconazole). Analgesia was induced with 100 ml of infulgan, and coagulation support was provided with fresh frozen plasma and 15,000 IU of heparin.

Carbamazepine and other suspected offending drugs were discontinued immediately. The patient was closely monitored for any signs of complications and received appropriate interventions when necessary.

### Outcome

**Figure 5:**
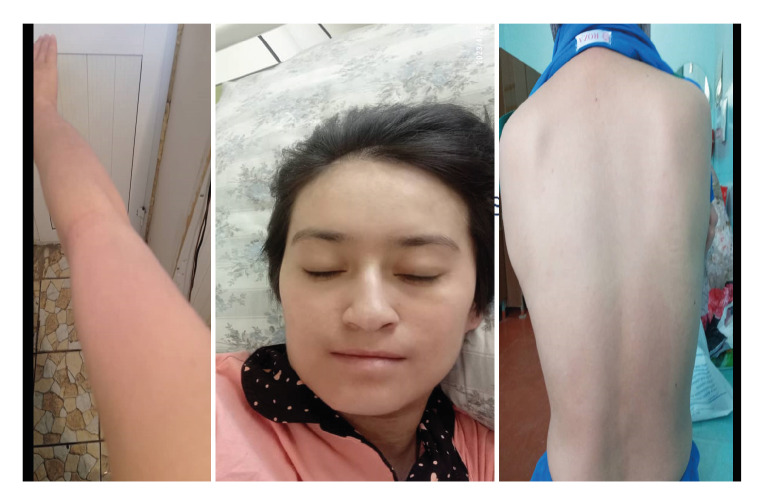
Clinical improvement of the patient with re-epithelialization and resolution of systemic symptoms.

The patient's clinical status progressively improved throughout hospitalization. The cutaneous manifestations commenced a healing process, and the mucosal erosions progressively ameliorated. Laboratory tests indicated a notable enhancement in hemoglobin concentration, platelet count, and hepatic function. After three weeks in the intensive care unit, the patient was discharged and transferred to a general ward for ongoing management and rehabilitation. Clinical recovery was characterized by re-epithelialization and resolution of systemic manifestations (figure 5).

### Ethical approval

The University Ethical Committee at the International Medical Faculty, Osh State University, approved the study for publication (No. IMFRC/case report/19/2024). Written informed consent was signed by the patient for the publication of this case report, including all clinical data and accompanying photographs with identifiable facial features.

## Discussion

Toxic epidermal necrolysis is a rare but potentially fatal condition characterized by widespread epidermal detachment and mucosal membrane involvement. Early identification and prompt intervention are imperative for enhancing patient prognosis. In the present case, the patient's clinical manifestations -encompassing the prodromal phase, epidermal detachment, and mucous membrane involvement- aligned with the diagnostic criteria for toxic epidermal necrolysis.

The intricate issue concerning genetic predispositions contributing to drug hypersensitivity has been extensively analyzed across different demographic groups and ethnicities. In the Han Chinese population, Chung *et al.* established a noteworthy correlation between HLA-B*15:02, Stevens-Johnson syndrome, and carbamazepine ^15^. An odds ratio of 25,04 underscored a significant relationship between antiepileptic medications and severe adverse reactions within a comparable ethnic cohort of Hong Kong Han Chinese ^16^. Furthermore, an additional investigation in Thai subjects corroborated the predisposition of individuals with the HLA-B*15:02 allele to carbamazepine-induced reactions ^17^.

Consequently, within the large-scale European RegiSCAR study, HLA-B genotyping was performed in patients who had experienced severe cutaneous adverse reactions to carbamazepine, allopurinol, and three other high-risk medications (sulfamethoxazole, lamotrigine, and NSAID of the oxicam class). The RegiSCAR findings demonstrated that HLA-B*15:02 is not a reliable biomarker for toxic epidermal necrolysis induced by carbamazepine, sulfamethoxazole, lamotrigine, or oxicam-type NSAID since it does not sufficiently explain the etiology of the disease in European populations ^18^^,^^19^. These results suggest that the genotype HLA-B*15:02 does not function as a population-independent marker for toxic epidermal necrolysis among individuals exposed to carbamazepine.

In this case, the immediate cessation of carbamazepine was vital for the effective management of toxic epidermal necrolysis. This fact underscores the critical need to identify the causative pharmacological agent and promptly discontinue its administration. The withdrawal of the medication played a fundamental role in attaining a favorable clinical outcome, a finding consistent with the results of Schneck *et al.,* who reported enhanced survival rates following the swift discontinuation of the causative drug ^12^.

Supportive care constitutes the cornerstone of toxic epidermal necrolysis management in the absence of universally accepted therapeutic guidelines. Its key elements include wound management, analgesia, and infection prevention. The objective of wound care is to avert further epidermal detachment and promote re-epithelialization. Trent *et al.* highlighted the significance of gentle wound cleansing, the use of non-adherent dressings, and regular monitoring for signs of infection to mitigate secondary complications and promote wound healing ^20^.

Effective pain management is paramount in toxic epidermal necrolysis due to the severe discomfort experienced by patients. Opioids and NSAID are frequently used to achieve analgesia, an application validated by Wolkenstein *et al.* among patients suffering from toxic epidermal necrolysis ^6^.

Infection prevention constitutes another critical component of supportive care in toxic epidermal necrolysis, as the denuded dermis is highly susceptible to bacterial colonization. In patients at increased risk, regular monitoring for early signs of infection and prompt initiation of prophylactic antibiotics can significantly mitigate complications. Trent *et al.* emphasized the crucial role of infection prevention in the therapeutic approach for this condition ^18^.

The use of systemic corticosteroids, intravenous immunoglubulin, and other immunomodulatory therapies in the management of toxic epidermal necrolysis sparks ongoing debate within the medical field. While some investigations postulate that the prompt administration of systemic corticosteroids may lead to a decrease in mortality and morbidity, other studies present contradictory evidence. Mockenhaupt *et al.* assessed the impact of systemic corticosteroids on toxic epidermal necrolysis and observed a decreased mortality rate when they were administered at an early stage of the disease ^10^.

Intravenous immunoglobulin has been utilized in the therapeutic regimen for toxic epidermal necrolysis, although its clinical efficacy remains indeterminate. Bachot *et al.* executed a randomized controlled trial comparing the administration of intravenous immunoglubulin against supportive care alone. They found a statistically significant reduction in mortality, along with improved clinical outcomes in the intravenous immunoglubulin-treated group ^11^.

## Conclusion

Toxic epidermal necrolysis represents a rare and potentially fatal dermatological condition often linked to adverse drug reactions. Early disease recognition, swift cessation of the causative pharmacological agent, and intensive supportive care are imperative for the effective management of the disease. Further investigations into genetic predisposition associated with the offending drugs are urgently needed to identify immunogenetic biomarkers that may enhance comprehension and guide management of this uncommon condition.
